# The end-of-treatment process in medically assisted reproduction: a qualitative study of healthcare professionals’ views

**DOI:** 10.1080/26410397.2025.2494412

**Published:** 2025-04-16

**Authors:** Federica Bonazza, Lidia Borghi, Sara Molgora, Elena Vegni, Daniela Leone

**Affiliations:** a PhD Student, Department of Psychology, Catholic University of Milan, Milan, Italy.; b Psychologist, Unit of Clinical Psychology, San Paolo Hospital, Milan, Italy; c Professor, Department of Psychology, Catholic University of Milan, Milan, Italy; d Professor, Department of Health Sciences, University of Milan, Milan, Italy; Psychologist – Chief of the Unit, Unit of Clinical Psychology, San Paolo Hospital, ASST Santi Paolo e Carlo, Milan, Italy; e Psychologist, Unit of Clinical Psychology, San Paolo Hospital, ASST Santi Paolo e Carlo, Milan, Italy; Lecturer, Department of Health Sciences, University of Milan, Milan, Italy

**Keywords:** medically assisted reproduction, end-of-treatment, decision-making process, shared decision making, physician–patient relationship

## Abstract

In the medically assisted reproduction (MAR) pathway, one of the most complex phases is the end of the treatment. Unlike other medical contexts, there is no biological endpoint in the MAR setting. This absence makes the decision to end MAR treatment extremely challenging for both patients and healthcare professionals. Accordingly, our research aimed to examine the process related to the end of MAR treatment, as devised by healthcare professionals. Our sample included physicians, biologists, and psychologists aged *≥*18 years with specialised training in assisted reproduction. Data were collected through four focus groups (in February–May 2023), focusing on the topic of the end of treatment (EoT) and its definition. Data were collected and analysed according to the principles of Grounded Theory. The findings shed light on the attributes and components related to the end of the treatment process. The central category “the end of treatment” consists of a definition of what is considered the end of treatment and the associated decision-making process. In the phase leading up to the EoT, the process is influenced by contextual and proximal factors, which interact and influence each other. To cope with and manage the EoT, healthcare providers adopt spontaneous strategies that lead to positive or negative outcomes. End-of-treatment management is a key facet of clinical practice. This contribution increased knowledge about EoT and highlighted healthcare professionals’ perspectives, which should be considered for the implementation of best practice points and respect for patients’ rights to the highest attainable standard of mental and physical health.

## Plain language summary

One of the most complex phases of the Medically Assisted Procreation (MAR) pathway is the end of treatment. Although this issue is fundamental, to date there is a lack of data on it. The research question of this study was: “How do healthcare professionals define and manage the end of MAR treatment?” Physicians, psychologists, and biologists took part in four focus groups. These groups focused on the topic of ending treatment, its definition, and management. We applied the Grounded Theory methodology to analyse the focus group transcripts. This theory allows us to explore an unexplored phenomenon and build a theory from the data. The end of treatment occurs when patients terminate MAR treatments. It is influenced by contextual and proximal factors. Through different strategies, it leads to negative or positive outcomes. End-of-treatment management is a key facet of clinical practice. These findings may improve the implementation of best practice points.

## Résumé

Dans le parcours de procréation médicalement assistée (PMA), l’une des phases les plus complexes est la fin du traitement. Contrairement à d’autres contextes médicaux, il n’y a pas de point final biologique dans le contexte de la PMA. Cette absence rend la décision de mettre fin à la PMA extrêmement difficile tant pour les patients que pour les professionnels de santé. Ainsi, notre recherche visait à examiner le processus relatif à la fin du traitement de PMA, tel que conçu par les professionnels de santé. Notre échantillon comprenait des médecins, des biologistes et des psychologues âgés de 18 ans ou plus ayant une formation spécialisée en procréation assistée. Les données ont été recueillies lors de quatre groupes de discussion (en février-mai 2023), en se centrant sur le thème de la fin du traitement et sa définition. Les données ont été collectées et analysées conformément aux principes de la théorie ancrée (*Grounded Theory*). Les résultats ont mis en lumière les attributs et les composants relatifs à la fin du processus de traitement. La catégorie centrale « la fin du traitement » consiste en une définition de ce qui est considéré comme la fin du traitement et du processus de prise de décision associé. Dans la phase menant à la fin du traitement, le processus est influencé par des facteurs contextuels et proximaux, qui interagissent et s’influencent mutuellement. Pour faire face à la fin du traitement et la gérer, les prestaires de soins de santé adoptent des stratégies spontanées qui aboutissent à des résultats positifs ou négatifs. La gestion de la fin du traitement est une facette clé de la pratique clinique. Cette contribution a permis de mieux connaître la fin du traitement et éclairé le point de vue des professionnels de santé, dont il faut tenir compte pour la mise en œuvre des meilleures pratiques et le respect du droit des patients au meilleur état de santé physique et mentale qu’ils soient capables d’atteindre.

## Resumen

En el proceso de reproducción con asistencia médica (RAM), una de las fases más complejas es el final del tratamiento. A diferencia de otros contextos médicos, el ámbito de RAM no tiene punto final biológico. Debido a esta ausencia, la decisión de terminar el tratamiento RAM es sumamente difícil tanto para pacientes como para profesionales de salud. Por consiguiente, nuestra investigación se propuso examinar el proceso relacionado con el final del tratamiento RAM, tal como concebido por profesionales de salud. Nuestra muestra consistió en médicos, biólogos y psicólogos de ≥18 años, con formación especializada en la reproducción asistida. Se recolectaron datos por medio de cuatro grupos focales (entre febrero y mayo de 2023), centrados en el tema del final del tratamiento (FdT) y su definición. Los datos fueron recolectados y analizados según los principios de la Teoría Fundamentada. Los hallazgos arrojan luz sobre los atributos y componentes relacionados con el final del proceso de tratamiento. La categoría central “el final del tratamiento” consiste en la definición de lo que es considerado como el final del tratamiento y en el proceso asociado de toma de decisiones. En la fase previa al FdT, el proceso es influenciado por factores contextuales y proximales, que interactúan y se influyen entre sí. Para abordar y manejar el FdT, el personal de salud adopta estrategias espontáneas que producen resultados positivos o negativos. El manejo del final del tratamiento es una faceta clave de la práctica clínica. Esta contribución aumentó el conocimiento sobre el FdT y destacó las perspectivas de profesionales de -salud, que deberían tomarse en consideración para la aplicación de los puntos de prácticas óptimas y respeto por el derecho de pacientes al más alto nivel posible de salud mental y física.

## Introduction

End-of-treatment (EoT) issues frequently arise in the medically assisted reproduction (MAR) context. In medicine, EoT is defined as the phase when a patient has completed all planned interventions related to a particular medical condition and is no longer receiving any active therapy.^1^

Unlike other medical settings, in the MAR context, there is no clear biological endpoint, except when treatment stops due to pregnancy. The Ethics Committee of the American Society for Reproductive Medicine^2^ stated that “clinicians may refuse to initiate a treatment option they regard as futile or having a very poor prognosis.” The term “futile” refers to all those treatments that have a ≤1% chance of achieving a live birth, and “very poor prognosis” refers to those treatments for which the odds of achieving a live birth are very low but not inexistent (>1% to ≤5% per cycle). Boivin and colleague^3^ claimed that EoT occurs in MAR when “the chance of success is so low that it is in the couple’s best interest to discontinue the treatment.” They refer to circumstances when there is the possibility to continue treatment, but the likelihood of success is extremely low and the continuation of treatment may be physically and psychologically burdensome. Thus, the EoT could be established based on physical, relational, or psychological difficulties, as well as financial costs for both patients and healthcare professionals.^4^

The decision to end MAR treatment is extremely challenging for both patients and healthcare professionals; it is one of the most difficult issues in the doctor–patient relationship.^5,6^ These situations raise questions about who should decide to discontinue treatment. The decision-making process is often defined as a cognitive process, but emotional aspects (for patients and healthcare professionals) must also be considered. Feelings, values, and beliefs can influence this process, which becomes more complex when it induces an experience of loss and appears irreversible, as in the case of EoT.^7^

For healthcare professionals, there is a risk of overlooking the emotional implications of EoT, as the decision to end treatment in MAR does not seem to be emotionally comparable to an end-of-life situation or a disease with an unfavourable prognosis.^8^ Healthcare professionals often feel pressured by patients’ expectations and experience a range of emotions related to MAR treatment, from a sense of omnipotence to a sense of powerlessness.^6,8^ These emotions may manifest as performance anxiety and compulsive use of MAR treatments^9^ and may influence clinical decisions regarding EoT. Furthermore, the evaluation of financial costs often precedes and impacts clinical decisions regarding EoT. In the Italian context, there are important differences between public and private centres in terms of resources, availability, and regulations.

From the patients’ perspective, the decision to stop therapy is particularly complex because it requires the patients to formally renounce their biological parenting. Previous studies have amply demonstrated that ending treatment without success can trigger overwhelming emotions, such as shock, denial, anger, frustration, loss of control, and existential failure.^10^ When making decisions on EoT, patients’ reproductive rights must be considered, because they are a crucial and integral part of this decisional process. Patients have the right to knowledge, education, and the autonomy to make reproductive choices.^11^ They also have the right to achieve the best possible reproductive health. According to Italian law 219 of 2017, the clinical decision-making process must guarantee the patient’s autonomy, and provide all the information required to fully understand the clinical situation.

Although the complexity of EoT has been acknowledged, this phenomenon remains relatively unexplored in the literature. Undoubtedly, managing the EoT has become a key professional skill, and the decision-making process at the EoT phase is an essential facet of healthcare practice. Therefore, this study aims to examine the process related to the end of MAR treatment, as perceived by healthcare professionals, by looking at how it is handled in decision-making and communication practice.

## Methods

### Study design

Given the paucity of data on this phenomenon and its related process, a qualitative methodology was employed. The qualitative method allowed exploration of the EoT process by drawing on the experiences and perspectives of healthcare professionals. We have chosen Grounded Theory (GT) methodology^12^ to formulate an emergent theory of an unexplored phenomenon by adopting an inductive approach.^13^ The COnsolidated criteria for REporting Qualitative research (COREQ) checklist^14^ has been used to determine important components of the study methodology, analysis, and interpretations.

### Participants

In line with GT procedures, convenience and theoretical sampling was carried out. Theoretical sampling is a form of sampling not bound by the limits of *a priori* selection.^15^ Rather, it allows participants to be progressively recruited based on data collected to gain new and emerging insights. The end of sampling is based on the saturation of the emerging theory, when no further data is necessary.^16^

According to this strategy, healthcare professionals working in national MAR clinics were involved through a procedure of several steps. First, an online invitation describing the study and its objectives was sent from the principal investigator to all the chief physicians at the MAR clinics. The chief physicians then proposed the study to their colleagues. Participants were recruited based on the following criteria: (1) healthcare professionals (gynaecologists, biologists, psychologists, nurses, laboratory technicians) with specialised training in assisted reproduction and with at least two years’ experience in EoT; (2) understanding and speaking Italian fluently. To maximise data variation, healthcare professionals were enrolled based on a wide variety of socio-demographic characteristics (sex and geographical area), professional role, and years of experience in EoT. The healthcare professionals gave their written informed consent electronically and were then asked for socio-demographic information. No relationship was established prior to study commencement.

### Data collection

Data collection took place from February 2023 to May 2023. To investigate the phenomenon of the EoT process, focus groups were set up online using Microsoft Teams, with sessions lasting about an hour and a half. We decided to conduct focus groups to promote dialogue among participants and to elicit responses, and introspection that would frequently not surface in one-on-one interviews. Each focus group involved five to eight participants and was conducted by three psychologists and researchers (FB, LB, and DL). During the focus groups, there were no people present other than participants and researchers. A semi-structured focus group guide was developed to ensure that the key areas were explored and pilot-tested before the beginning of data collection. The interview guide was based on a literature review. The quality of the interview was checked by assessing questions’ clarity, pertinence, relevance, completeness with colleagues of the authors who worked in ART centres. These colleagues were then excluded from the study. Each focus group began with a statement of the purpose of the research, followed by a discussion of the definition of EoT in MAR and an exploration of experiences, strategies for management, and approaches. The researchers declared their role in this research project. Field notes were made during and after focus groups. The audio of the focus groups was recorded with participants’ consent and transcribed verbatim.

### Data analysis

Data analysis was independently carried out by two researchers (DL and FB), along with the collection of data for constant comparison.^17^ The transcript was analysed in accordance with grounded theory procedures, made up of three phases: open coding, axial coding, and selective coding. In the “open coding” phase, an initial line-by-line textual analysis of all the transcripts was conducted. The data were fitted into salient flexible open codes, which were labelled using the participants’ words. Coding was performed after each focus group to integrate the new data into the previously identified code system and to identify topics to be covered in subsequent focus groups.^18^ The second process of “axial coding” led to the progressive aggregation and condensing of codes into broader categories. This phase aims to identify the main directions, themes, and categories indicated by the totality of the data.^18^ The open coding and axial coding phases are recursive since the results of the analysis direct the data collection.

Once the saturation level is reached, the conclusive “selective coding” phase begins. This phase qualified the relationships among the categories by increasing abstractions. The final explanatory theory was formulated by determining categories and subcategories, elucidating the relationships between them, and identifying the central category that represents the organisational concept of the investigated process.^18,19^

Any disagreements emerging during the analysis process were discussed with other researchers of the team (SM, EV). All authors are psychologists with previous experience in the field of infertility. The team organised a workshop to present and validate the theoretical model. Consistent with theoretical guidelines,^20^ all participants were invited to provide their feedback at the workshop.

### Ethics

Informed consent was obtained electronically from all the participants. The study was conducted according to the guidelines of the Declaration of Helsinki and was approved by the Ethics Committee of the University of Milan (approved on 28th October 2022).

## Results

### Participants

A total of 13 Italian MAR clinics (5 public clinics, 2 private clinics, 6 private contracted clinics) were invited using a convenience, non-probabilistic sampling approach. Twenty-four healthcare professionals from eleven MAR clinics were involved in four focus groups (Focus Group (FG)1 = 8 participants; FG2 = 6 participants; FG3 = 5 participants; FG4 = 5 participants). The sample included 15 gynaecologists, 5 biologists, and 4 psychologists. Two clinics accepted the invitation, but the affiliated healthcare professionals were unavailable. Thirteen of the 37 professionals who were invited dropped out: 10 healthcare professionals did not show up in the focus groups, and 3 healthcare professionals withdrew because of unexpected job constraints. Participants’ socio-demographic and professional characteristics are reported in Table 1. The participants were mainly females (79.2%), with a mean age of 53 (SD = 11) years and an average of 20 (SD = 11) years of clinical experience. Most participants worked in northern Italy (45.8%) and central Italy (37.5%).

**Table 1.** Socio-demographic and professional characteristics of the study population

Characteristics	Healthcare professionals *N* = 24
Age	
Mean; SD	53; SD = 11
Range	37–70
Race/ethnicity, Caucasian (%)	100%
Gender	
Female (%)	79.2%
Male (%)	20.8%
I’d rather not answer (%)	0%
Years of experience	
Mean; SD	20; SD = 11
Range	3–40
0 ≤ 30	17 (70.8%)
>30	7 (29.2%)
Working context	
Public	9 (37.5%)
Private	3 (12.5%)
Private contracted	12 (50%)
Institutional location	
North Italy	11 (45.8%)
Central Italy	9 (37.5%)
South Italy	4 (16.6%)

### The end-of-treatment process

**Figure 1.**
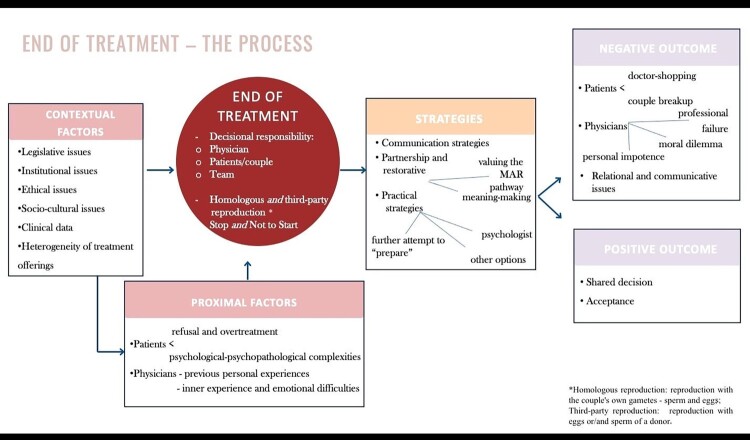
The end-of-treatment process in medically assisted reproduction

The overall EoT process, as recounted by MAR healthcare professionals, is shown in Figure 1. The EoT process evolves in three main stages. In the pre-EoT phase, the process is influenced by contextual and subjective factors that interact and influence each other. The end of treatment, which is the core category, comprises two aspects: its definition and the decision related to it. To address the consequences of EoT, healthcare professionals employ spontaneous strategies, leading to two different types of outcomes: negative or positive.

The categories of the model are described below, along with some quotes drawn from the transcripts of the focus groups, given as examples. The quotes are followed by the number of the focus group from which they came, the identification number of the participant, gender, and her/his profession (e.g. FG1, P1, male, gynaecologist).

### End-of-treatment definition and the decision related to it

The end of treatment is the phase that determines the end of the MAR pathway. EoT occurs either on patients’ own initiative or at healthcare professionals’ recommendations. EoT arises when patients (1) terminate homologous reproduction (e.g. reproduction with the couple’s own gametes – sperm and egg) or (2) terminate third-party reproduction (e.g. reproduction using eggs or/and sperm of a donor). Participants emphasised the difference between these two scenarios. Participants claimed that if patients stop homologous reproduction but are thereafter open to third-party reproduction, it is inappropriate to talk about the end of the treatment.

*“The homologous end of treatment is actually not a true end because you can still conduct a subsequent therapy that could be third-party, so if we want to talk about total end of treatment we still have to differentiate between homologous and third-party.”* (FG1, P2, female, gynaecologist)

Thus, participants consider third-party reproduction as a new opportunity, which postpones or avoids the EoT phase. When defining the EoT, some participants made a distinction between it and the state categorised as “not to start.” The “not to start” state includes those particular and severe clinical conditions in which treatment is not even begun as it is unlikely to be successful or there are ethical and moral complexities. *“Here there was an issue of risk to the woman’s life, and risk to the child (…) so I mean, this is not management, this is a non-start”* (FG1, P6, male, gynaecologist).

The EoT core category also concerns decisions related to the end of the MAR pathway. Decision-making responsibility may be taken by physicians, interdisciplinary teams, patients, or jointly by healthcare professionals and patients. Healthcare professionals reported an ideal condition in which the decision to end treatment is shared with the patients and multidisciplinary teams. However, participants claimed that given the lack of time, this ideal scenario rarely occurs. *“My view is that, in almost all cases, it is the couple that decides the end of the treatment. We don’t decide together”* (FG1, P6, male, gynaecologist). Sharing with patients and teams would be desirable, particularly in critical situations.

In situations in which decisions are not made jointly, physicians primarily rely on biological and medical data. *“(…) A couple has such a low success rate, (…) there is the moment when you have to say no to that couple, explain that it doesn’t make sense to keep repeating the treatments”* (FG1, P5, male, gynaecologist). Sometimes the decision was discussed by the entire interdisciplinary team: *“(…) There is the voice of the treating doctor who has managed the couple, there is the voice of the biologist, then there is the head doctor, the laboratory chief; so, everyone can express their opinion”* (FG1, P7, female, psychologist). Instead, when the decision is made by patients, it is mainly based on emotions, values, and treatment side effects. *“Abandonment by couples may occur for various reasons – one is that the burden on the couple is intolerable”* (FG1, P6, male, gynaecologist).

### Factors influencing decision-making

#### Contextual factors

Participants reported that the EoT process is impacted by outside variables resulting from the context in which the care pathway is established. These “contextual factors” include several aspects, such as legislation, institutional limitations, and ethical issues. Legislation can restrict access to care according to the patient’s age, previous medical interventions, and clinical examination results. Similarly, institutions may restrict the provision of clinical treatment; for instance, in Italy, not all MAR centres offer third-party reproduction. *“In the public service, the number of treatments is set by the region, so the end of treatment is when they have finished the number of cycles they could do, so that’s the limit”* (FG1, P6, male, gynaecologist). Moreover, participants took ethical considerations into account. Distributive justice value is applied to limit treatment so that everyone has access to available resources. *“The waiting list is getting longer and longer and so we have to somehow be or try to be fair”* (FG1, P5, male, gynaecologist).

Furthermore, a specific role was attributed to society and culture. Participants introduced “sociocultural factors.” In Italy, society and a family-centred culture may accentuate patients’ beliefs and reinforce discrepancies between the views of healthcare professionals and patients. Healthcare professionals may prioritise clinical needs, while patients may give priority to the goal of parenthood. The desire for pregnancy boosts patient persistence and optimism regarding outcomes. *“We are victims of wishful thinking* * … * *In the field of reproduction and reproduction-related pathology, there is less acceptance that we cannot go beyond a certain clinical limit”* (FG2, P4, male, gynaecologist).

The lack of a biological endpoint creates significant variability in proposals. Differences often arise due to the different priorities and values given to intervening factors (such as patient autonomy, patient beneficence). *“I mean there are doctors who see the futile as a treatment not to be done, those who,* * … * *maybe have a more bioethical view and say ‘but if the patient is informed, I will give them the treatment’”* (FG2, P5, female, gynaecologist). So, one physician would not initiate treatment if the probability of success is low, and another would proceed with a low-likelihood-of-success procedure because the patient, after receiving complete information, wants it.

#### Proximal factors

Participants introduced “proximal factors,” meaning elements related to the viewpoints of the individuals involved in the MAR procedure. From the patients’ point of view, the participants highlighted high pressure and demand. Even when physicians recommend discontinuing treatment, patients often reject the recommendation and insist on achieving the goal. *“(…) There are couples who don’t get off the chair and have no intention whatsoever of putting aside the idea of continuing* * … * *a kind of doggedness* (* … *)*”* (FG4, P1, female, biologist). Furthermore, patients’ psychological and psychopathological factors are known to influence the EoT process.

*“(…) the couple is so complex, not only from a medical point of view, but also from a socio-intellectual point of view (…). There are also such fragile and critical situations where we, as a group, also ask ourselves what we should avoid (…).”* (FG1, P5, male, gynaecologist)

Participants also referred to their own subjective issues, such as previous personal and professional experiences, emotional challenges, communication and relational difficulties. These factors may influence the EoT process. *“Because saying no so many times gets really tiring after a while (…)*. *The fact of declaring a bankruptcy (…) we failed in our attempt to help”* (FG1, P8, male, gynaecologist).

### Strategies employed by healthcare professionals

The participants reported implementing different strategies to cope with the EoT challenges. EoT disrupts the clinical pathway; therefore, healthcare professionals try to manage it through several different strategies. Some physicians emphasised the importance of offering patients clear and detailed explanations. Thus, *“the explanations must always be very clear and transparent, using simple language, which the couple can understand”* (FG3, P3, female, biologist). However, managing communication with the patients is challenging. *“It is a big communication problem, our inability was not to explain well what was going on (…)”* (FG1, P4, male, gynaecologist).

The participants used partnership and restorative strategies to express closeness and regret for not achieving the desired outcome. *“The winning strategy is to communicate some involvement in the problem, that is, to try to make it clear that you too are humanly sorry* * … ”* (FG1, P8, male, gynaecologist). In this approach, healthcare professionals valued the MAR pathway and the efforts made, encouraging a redefinition of identity and a new pathway’s meaning. *“One of the things that I see working to a certain point is valuing the fact that they really did everything they had to do* * … * *I often say ‘You really have done everything you could* * … ’”* (FG1, P6, male, gynaecologist).

In addition to these strategies, participants identified pragmatic approaches. For example, physicians occasionally offer a final round of treatment, making it clear that this is the last attempt. This strategy could help patients to cope with feelings of loss that may arise during the EoT. *“Be able to say: ‘we have tried together, that’s feasible, let’s do one more cycle but also be prepared that then the programme ends.’ So, to anticipate the end* * … ”* (FG1, P5, male, gynaecologist). Sometimes, participants considered third-party reproduction as an additional alternative. *“It is clear that the addition of third-party has given us a plan B that clearly postpones an end of treatment”* (FG2, P5, female, gynaecologist). Other pragmatic strategies adopt a more advisory nature, introducing patients to the possibility of choosing childlessness or adoption as a solution. *“I propose this possibility of a childless life, but I don’t think gynaecologist do that. I say ‘have you thought that your life could also be different?’ And so, this is a time for reflection”* (FG4, P1, female, biologist). Moreover, some participants advised seeking treatment from a psychologist to reprocess the loss following the EoT. *“‘I strongly recommend that you contact our psychologist or at least have her follow you through this difficult time,’ I mean do not ‘consider it,’ just ‘do it’”* (FG2, P5, female, gynaecologist).

### Outcomes

The EoT process leads to outcomes defined by participants as “negative” or “positive.”

#### Negative outcomes

Participants define outcomes as “negative,” when they involve an adverse impact, i.e. the onset of a negative experience. Negative experiences may concern the couple’s relationship, the doctor–patient relationship, and/or the healthcare professionals’ emotional experiences.

As for couples, the participants explained that giving up biological pregnancy could break the relational balance. *“The moment you give them news like this, you break their homeostasis (…) because they know that they would no longer recognise themselves as a couple”* (FG2, P3, female, psychologist). At the same time, a phenomenon known as “doctor shopping” can occur, where patients start to move between institutions in search of a physician who allows them to continue their treatment.

In terms of healthcare professionals–patients’ relationships, negative outcomes can include communication and relational problems. In particular, conflicts often occur when patients want to continue treatment but physicians recommend stopping it. *“Sometimes you really enter the limit of conflict to say stop (…) That’s difficult* * … ”* (FG1, P8, male, gynaecologist). A sense of helplessness also emerged when professionals considered themselves unprepared to handle EoT conversations. *“The fundamental problem is that doctors are basically not trained to communicate this news”* (FG2, P3, female, psychologist).

Negative outcomes can also influence the inner lives of healthcare professionals. The weight of responsibility can cause distress; the actions, choices, and roles of healthcare providers can be called into question.

#### Positive outcomes

The participants provided a definition of “positive” outcomes. A positive outcome does not coincide with pregnancy, as it can also occur in the event of treatment failure. It can be achieved if the end of treatment is well managed by patients and healthcare professionals, that is, if there is good agreement with the decision. Thus, patient acceptance of the EoT represents a positive outcome. *“The possibility of placing an internal limit, that is that couples, during the work we do together, activate within themselves the possibility of setting a limit* * … ”* (FG3, P4, female, psychologist). Another way to achieve positive outcomes is to share the decision between healthcare professionals and patients. Due to the challenges of implementing a shared decision-making process, a decision made jointly by patients and an interdisciplinary team is considered successful. *“Communicating with them in person with the patient, with the couple, in some situations it was much easier”* (FG4, P1, female, biologist).

## Discussion

The main finding of this study was the development of a theoretical model of the EoT process. EoT occurs when patients interrupt MAR treatment either at their own choice or at the discretion of healthcare professionals. EoT arises when patients terminate homologous reproduction or terminate third-party reproduction. According to participants, we cannot discuss EoT if a patient discontinues homologous reproduction treatment but is open to third-party reproduction. Third-party reproduction is another treatment option.

In the core category EoT, participants placed a high value on decision-making responsibilities. EoT decision-making in the MAR context differs from those in other medical contexts. In many clinical settings, such as Intensive Care Units or Palliative Care, guidelines help identify EoT and guide clinical choices and practice.^21^ Unlike in other medical contexts, there is no biological endpoint in the MAR setting. Some institutions have established guidelines to indicate when MAR treatments should be considered futile and discontinued;^2^ however, these criteria are not always applied. Clinical decisions regarding discontinuation are often highly variable and arbitrary with an elevated risk of misalignment among professionals. In the MAR context, physicians, interdisciplinary teams, and patients are stakeholders in the decision-making process. However, our results suggested that decision-making responsibility often comes down to a single stakeholder, without sharing it in interdisciplinary teams and/or with patients. For about two decades, a patient-centred medicine model and the related concept of shared decision-making (SDM) have been gaining ground.^22^ SDM has been defined as a process in which physicians and patients work together to define treatment goals and share information about preferred options and outcomes with the aim of reaching mutual agreement on the best course of action.^23^ Although SDM has been recommended as an ideal model in medical practice, our findings suggest that it may be difficult to apply in the MAR context. The inapplicability of SDM is often due to several factors, such as a lack of time, a lack of agreement between the clinician’s perspective and that of the patients, complex patient characteristics, excessive pressure to achieve the goal, and a lack of common discussions. Moreover, previous studies have claimed that healthcare professionals may perceive SDM as a threat to professional autonomy.^24^ In summary, our results revealed a discrepancy between the perspectives of healthcare professionals and patients, which makes it difficult to enforce a shared agreement on the best course of action.

This discrepancy is often influenced by contextual and proximal factors. The EoT process is influenced by contextual factors, including legislation, institutional rules, and ethical issues. Contextual factors create circumstances in which MAR treatment can occur. They act as both aids and constraints in the decision-making process. In the absence of a defined endpoint, these factors play a crucial role in guiding decisions and providing an institutional limit where a biological one is lacking. Regarding ethics, there is a dual dimension related to ethical consciousness and professional ethics.^25^ Participants emphasised professional ethics by citing values of the medical profession, such as distributive justice, beneficence (that is the physician’s duty to act with respect for the patient’s welfare), and the patient’s right to autonomy in decision-making. However, ethical consciousness seems to be crucial. Healthcare professionals provided examples of clinical scenarios in which they had to decide which course of action best suited their ethical idea of the patient’s well-being.

The EoT process is also influenced by several proximal variables. The participants reported significant difficulties in establishing and maintaining satisfactory relationships with their patients. Patients are described as critical, demanding individuals with unrealistic expectations, leading to insistence on their treatment. These findings echo the wishful thinking perspective and previous literature that has extensively highlighted patients’ experiences during treatment.^10,26,32^

The management of EoT requires strategies. Some of these focus on communication, whereas others hinge on the relationship between healthcare professionals and patients. The literature provides guidelines for breaking bad news in medicine.^27^ Currently, the SPIKES protocol is widely recognised and provides guidelines for effective communication of medical bad news. The protocol consists of six steps to ensure the creation of an appropriate setting, collection of patient information, communication of medical information, and long-term planning.^28^ Recent studies have acknowledged the specificity of the MAR context and revised Buckman’s SPIKES protocol.^5,6^ However, communication of EoT decisions in the MAR context requires further attention, considering patients’ experiences, difficulties, and potential resources in this phase.^30^ Physicians presented training demands that must be promptly addressed. As the main communicators in charge of EoT-related communications, physicians believe that they must enhance their communication skills to be more successful in breaking bad news about EoT.

Furthermore, relational strategies are crucial for valorisation of the MAR pathway. Sometimes, healthcare professionals encourage patients to appreciate what they have accomplished, before arriving at the EoT. Convincing patients that everything that could have been done has been done mitigates the risk of regret and is often vital for the doctor–patient relationship and patient’s well-being. Healthcare professionals may decide not to interrupt the care pathway, and instead assist patients in considering alternative plans, including third-party reproduction or adoption. Third-party reproduction can be seen as an alternative treatment that allows healthcare professionals to offer additional interventions and postpone or avoid EoT. Our results showed that switching to third-party reproduction may be a way to reprocess grief over previous failures and encourage hope for a new form of pregnancy. The transition from homologous to third-party reproduction is frequently regarded as nearly automatic in MAR clinics. Nonetheless, patients must be given the opportunity to consider their options and the time to become familiar with this alternative method of reproduction. These issues may have resulted from the peculiarities of the Italian context, where third-party reproduction was only introduced as recently as 2014.

The EoT process results in an outcome that is classified as positive or negative. It is important to recognise that a positive outcome does not coincide with pregnancy, as it can be achieved even in unsuccessful situations. EoT often results in conflicts in the doctor–patient relationship due to unfulfilled patient expectations. Thus, if a satisfactory relationship with the patient is maintained and there is agreement on treatment goals and clinical decisions, this is considered a positive outcome. Participants also consider the implementation of SDM to be a successful outcome. Finally, another successful outcome is the patient’s acceptance of the EoT. It is important to emphasise that EoT need not be characterised solely by negative emotions. Previous studies have shown that EoT is sometimes associated with a sense of relief^26,31^ if there is proper guidance and support from healthcare professionals.

The results of the EoT process can also be seen as negative. As widely highlighted in the literature, when MAR treatment is discontinued, patients must accept that biological pregnancy can never be achieved.^10,32^ This painful awareness can add psychological suffering to the stress already caused by previous failed attempts.^10,32^ Although some patients may experience a sense of relief, EoT is a loss that affects their psychological well-being and may therefore require psychological support.^29^

Negative outcomes can also impact the professionals’ emotional experience. EoT may lead to a sense of failure among both patients and healthcare professionals. The possibility of giving life creates an illusion of omnipotence,^6^ but failure can demand an entire reformulation of self-image, given the perceived discrepancy between the ideal and reality. These findings are in line with those of previous studies,^8,9^ suggesting that the perception of professional failure may lead to persistent and compulsive use of MAR techniques.

This contribution can help healthcare professionals to implement guidelines for managing the EoT procedure (Table 2). Our findings showed a clear difficulty in applying SDM. This difficulty appears to be due to the discrepancy between the patient’s perspective and that of the healthcare professionals. Patients often report feeling abandoned during and after the decision-making process,^29^ highlighting the need for guidance at this complex stage. Healthcare professionals should also prioritise patient rights. As argued by Chan and Ho,^11^ it is crucial that the patient’s right to knowledge and information is enforced. Patients should have all the information about their clinical situation and treatments they may be undergoing. It is also important to respect the right to autonomy in clinical decisions regarding their health.^11^ Therefore, it is essential for healthcare professionals to explore patients’ experiences and expectations, to understand their values and preferences and facilitate the implementation of SDM. Dedicated decision-making counselling sessions or discussions about the possibility of failure should become a part of routine care.^30,33^

**Table 2.** Research-informed recommendations to support clinicians in managing the EoT process

Fill the communication gap between clinicians and patients by being aware of what patients expect from these discussions (see Ref.^30^) Do not reduce the EoT decision and communication to the last conversation with the patient Promote the development of explicit and shared evidence-based policies to guide decisions about initiating or stopping treatment Share the responsibility of the EoT decision in teamwork, especially in complex situations Consider the role of mental health professionals in supporting both patients and staff Reframe the goal of infertility care, focusing not only on the treatment of infertility but also on the patients’ well-being during the whole infertility pathway

As for communication, findings showed perceived inadequacy among healthcare professionals; thus, training and evidence-based policies may provide a guide.^34^ Specifically, consideration must be given to the intricacy of the variables, the communication gap caused by biases, and the role of teamwork, which is crucial, particularly under dire circumstances.

To the best of our knowledge, no previous study has examined the end of MAR treatment by drawing on the experiences and perspectives of healthcare professionals. However, this study had some limitations. A limitation of this study relates to convenience sampling. Convenience sampling is based on the accessibility and availability of the participants. The sample did not include nurses and technical staff due to the unavailability of invited healthcare professionals. Our results showed a shared perspective across all professions involved or called upon; however, future studies could enrich the perspective with the involvement of nurses, technicians, etc. Furthermore, the participants were predominantly female which reflects the generally low prevalence of male staff in the sector. These factors may have influenced our findings. Moreover, the qualitative nature of the study leads to limitations in the transferability of findings. Cultural bias may have an impact since the perspective of healthcare professionals can be influenced by their context. Our state has specific peculiarities in terms of regulations, treatment availability, and healthcare provision. Finally, a desirability bias should be taken into account, as participants may have found it difficult to share divergent views.

This study excluded patients’ perspectives; thus, future studies should explore their lived experiences concerning the end of treatment and the related processes. Despite these limitations, the qualitative approach used in this study allowed us to thoroughly explore and understand the process leading to treatment discontinuation. Moreover, we endeavoured to ensure the reliability of our qualitative data.^35^

## Conclusions

This contribution showed a theoretical model of the end-of-treatment process in the MAR context. Findings highlighted the dynamics and factors associated with the end of the treatment process, revealing healthcare professionals’ perspectives. These perspectives should be considered for the implementation of best practice points.

## Supplementary Material

Additional material: List of the Italian regulations regarding access to medically assisted reproduction treatment.

## Data Availability

Data will be shared on request to the corresponding author.

## Provenance statement

This paper was not commissioned and went through an external peer review.
