# Supplementary material for: The end-of-treatment process in medically assisted reproduction: a qualitative study of healthcare professionals’ views
Source: Sex Reprod Health Matters. 2025 Apr 16;33(1):2494412. doi: 10.1080/26410397.2025.2494412 (PMC12086948; doi:10.1080/26410397.2025.2494412)
Supplement: Additional material: List of the Italian regulations regarding access to medically assisted reproduction treatment. [file ZRHM_A_2494412_SM0724.docx]

The manuscript aims to explore the experience and perspective of healthcare providers regarding the end of MAR treatment. These perspectives are influenced by contextual factors (which also appear in the results); however, they cannot be totally attributed to them. 
However, to make the paper easier to read, the Italian regulations regarding access to medically assisted reproduction treatment are listed below.

19 February 2004, n. 40

**"** **Regulations on Medically Assisted Reproduction"**

ART. 4.

(Access to techniques).

1. The use of medically assisted procreation techniques is permitted only when it is ascertained that it is impossible to otherwise remove the causes preventing procreation and is in any case limited to cases of unexplained sterility or infertility documented by medical act as well as to cases of sterility or infertility from a cause ascertained and certified by medical act.

ART. 5.

(Subjective requirements).

1. Without prejudice to the provisions of Article 4, Paragraph 1, medically assisted procreation techniques may be accessed by adult couples of different sexes, married or cohabiting, of potentially fertile age, both living.

ART. 12.

(General prohibitions and penalties).

6. Anyone who, in any form, carries out, organizes, or publicizes the commercialization of gametes or embryos or surrogacy of motherhood shall be punished by imprisonment of three months to two years and a fine of 600,000 to 1 million euros.
